# Review of *Dolichostyrax* Aurivillius (Cerambycidae, Lamiinae) in Borneo, with descriptions of three new genera and the first case of (ovo)viviparity in the long-horned beetles

**DOI:** 10.3897/zookeys.587.7961

**Published:** 2016-05-10

**Authors:** Radim Gabriš, Robin Kundrata, Filip Trnka

**Affiliations:** 1 Department of Ecology & Environmental Sciences, Faculty of Science, UniversityPalacký University, Šlechtitelů 27, 783 71, Olomouc, Czech Republic; 2 Department of Zoology, Faculty of Science, UniversityPalacký University, 17. listopadu 50, 771 46, Olomouc, Czech Republic

**Keywords:** Coleoptera, diversity, endemism, hot-spots, Malaysia, Morimopsini

## Abstract

We reviewed the species of genus *Dolichostyrax* Aurivillius (Cerambycidae: Morimopsini) from Borneo, which included the redescriptions of two species – *Dolichostyrax moultoni* Aurivillius, 1911 and *Dolichostyrax longipes* Aurivillius, 1913, with the first female description for the latter. After the examination of the additional material previously identified as *Dolichostyrax*, we described three new genera – *Borneostyrax* **gen. n.**, *Microdolichostyrax* **gen. n.**, and *Eurystyrax* **gen. n.** *Borneostyrax cristatus* **sp. n.** was described based on the male and female specimens, whilst *Microdolichostyrax hefferni* **sp. n.**, *Microdolichostyrax minutus* **sp. n.** and *Eurystyrax nemethi* **sp. n.** are known only from females. All studied species are distributed in the mountain regions of Sabah, with the exception of *Dolichostyrax moultoni* from Sarawak. An identification key to the genera of Bornean Morimopsini and species of *Dolichostyrax*, *Borneostyrax* **gen. n.**, *Microdolichostyrax* **gen. n.** and *Eurystyrax* **gen. n.** is provided and their distributions and intraspecific morphological variability are discussed. The short and wide ovipositor, loss of spermatheca, and presence of large larvae without apparent eggbursters inside the female abdomens indicate the presence of (ovo)viviparity in *Borneostyrax* **gen. n.** This is the first case of this rare phenomenon within Cerambycidae.

## Introduction

Long-horned beetles (Cerambycidae) with about 35,000 described species are the fifth largest beetle family in the world (Švácha and Lawrence 2014). Although they are widespread, well-known, easily recognized and intensively collected by both amateurs and professional entomologists, their classification is still not well understood. For example, Lamiinae forms by far the most species-rich cerambycid subfamily, however, almost nothing is known about their interrelationships. Many supraspecific taxa are only vaguely defined, and a complete revision of the tribal classification is deeply warranted (Ślipiński and Escalona 2013). Morimopsini is most probably a polyphyletic lineage currently containing about 200 species classified in 50 genera distributed mainly in the tropical areas of Africa and Asia (e.g., Breuning 1950, Sudre and Teocchi 2002, Vitali and Menufandu 2010, Nearns et al. 2015, Tavakilian and Chevillotte 2015, Weigel 2015). Three small genera are reported from Borneo: endemic *Anexodus* Pascoe (two species) and *Pantilema* Aurivillius (monotypic), and *Dolichostyrax* Aurivillius (two species; with remaining congeners known also from Java and Sumatra) (Breuning 1950). Members of these genera are flightless and inhabit the leaf litter in rain forests. They are only rarely collected and there is absolutely no information on their morphological inter- and intraspecific variability, immature stages, distribution, relationships, biology, and ecology. Breuning (1950) made a generic and species identification key and since then, no attention was paid to the Bornean Morimopsini except for an isolated description of a new *Anexodus* by Sudre (1997).

Herein, we review the *Dolichostyrax* species in Borneo, which includes the redescriptions of *Dolichostyrax moultoni* Aurivillius, 1911 and *Dolichostyrax longipes* Aurivillius, 1913 and the descriptions of three new genera closely related to *Dolichostyrax*. For the first time, male and female genitalia are investigated and the identification key is provided for the Bornean Morimopsini.

## Material and methods

The study is based on adult semaphoronts of both sexes. Before the investigation of the external morphological characters, specimens were cleaned from a crust of dirt in a sonicator, following the method of Harrison (2012). The genitalia of both sexes were briefly kept in hot 10% KOH, dissected, transferred to glycerol and subsequently photographed using a Zeiss Discovery.V12 with ZEN software. The line illustrations were derived from the photographs. All dissected parts were mounted on the separate cardboards using the DMHF (Dimethyl Hydantoin Formaldehyde) resin and pinned with specimens. The measurements of taxonomically relevant morphological structures were taken with a measuring tool in ZEN software. The following abbreviations were used: BL – body length, measured from the fore margin of head to the apex of elytra; BW – maximal body width. Data from the locality labels are cited verbatim. A slash (/) is used to separate lines on the same label and a double slash (//) is used to separate different labels on the pin. The morphological terminology follows those of Ślipiński and Escalona (2013) and Švácha and Lawrence (2014).

### Depositories

HNHM
Hungarian Natural History Museum, Budapest, Hungary (O. Merkl, T. Németh) 


NHRS
Swedish Museum of Natural History, Stockholm, Sweden (J. Bergsten) 


PCDH
 personal collection of Daniel J. Heffern, Houston, TX, USA 


PCJC
 personal collection of Jim Cope, San Jose, CA, USA 


PCLB
 personal collection of Larry G. Bezark, Sacramento, CA, USA 


## Taxonomy

### Genus Dolichostyrax

Taxon classificationAnimaliaColeopteraCerambycidae

Aurivillius, 1911

Dolichostyrax Aurivillius, 1911: 194.
Dolychostyrax Breuning, 1950: 162 (incorrect subsequent spelling).

#### Type species.

*Dolichostyrax moultoni* Aurivillius, 1911.

#### Diagnosis.

*Dolichostyrax* differs from *Microdolichostyrax* gen. n. and *Eurystyrax* gen. n. by longer antennae (0.9–1.3 times as long as BL vs. 0.6–0.7, respectively), antennomere XI shorter than III (Figs 5, 16, 24, 31, 37, 44), relatively thinner antennomeres (antennomere III length/width ratio = 3.2–4.1 vs. 1.7–2.4, respectively), and metatarsomere III longer than metatarsomere I. *Borneostyrax* gen. n. differs from *Dolichostyrax* by bidentate mandibular apex (vs. unidentate; Figs 6, 52), elytra with tubercles forming distinct ridges (vs. rows of individual tubercles; Figs 2, 48, 60), distinct protrusions on apices of protibiae and mesotibiae along with tibial spurs 0-0-2 in males (vs. no protrusions and tibial spurs 2-2-2; Figs 8, 54), and terminal maxillary and labial palpomeres widened, flattened and truncate in males (vs. fusiform; Figs 7, 53).

**Figures 1–12.**
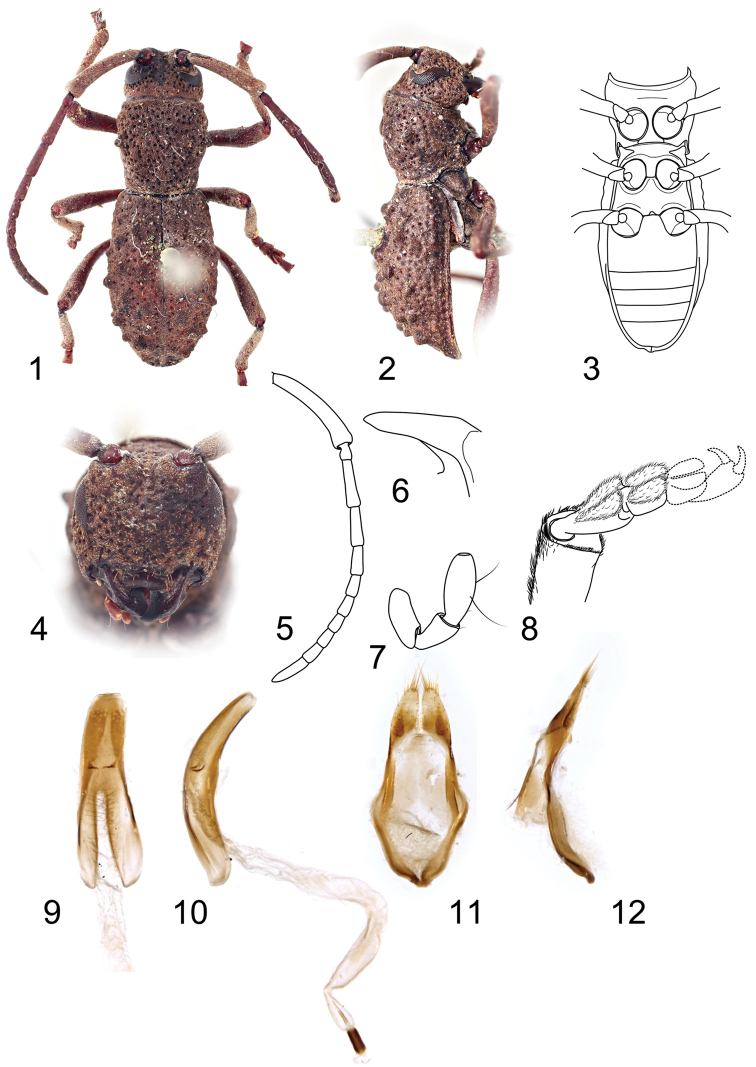
*Dolichostyrax moultoni* Aurivillius, holotype male: **1** Dorsal habitus **2** Lateral habitus **3** Ventral habitus **4** Head, frontal view **5** Antenna **6** Mandible apex **7** Apical maxillary palpomeres **8** Apex of protibia with protarsus **9** Penis, ventral view **10** Penis, lateral view **11** Tegmen, ventral view **12** Tegmen, lateral view. Not to scale.

**Figures 13–26.**
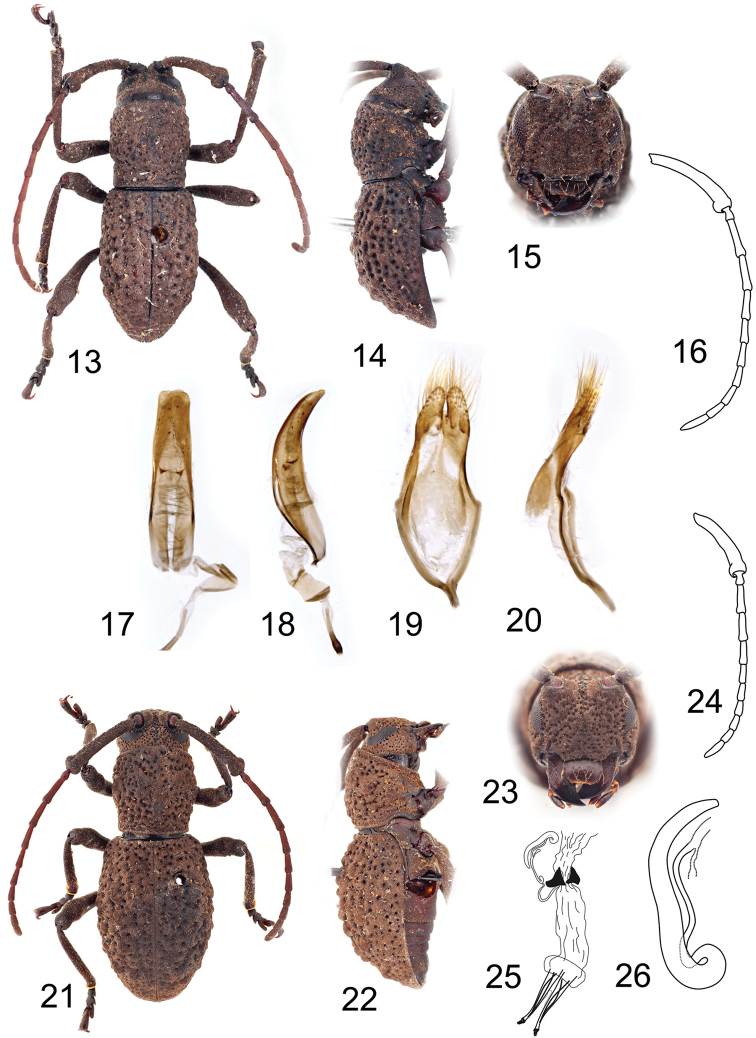
**13–20** *Dolichostyrax longipes* Aurivillius, holotype male: **13** Dorsal habitus **14** Lateral habitus **15** Head, frontal view **16** Antenna **17** Penis, ventral view **18** Penis, lateral view **19** Tegmen, ventral view **20** Tegmen, lateral view **21–26** *Dolichostyrax longipes* Aurivillius, female: **21** Dorsal habitus **22** Lateral habitus **23** Head, frontal view **24** Antenna **25** Reproductive system **26** Spermatheca. Not to scale.

**Figures 27–39.**
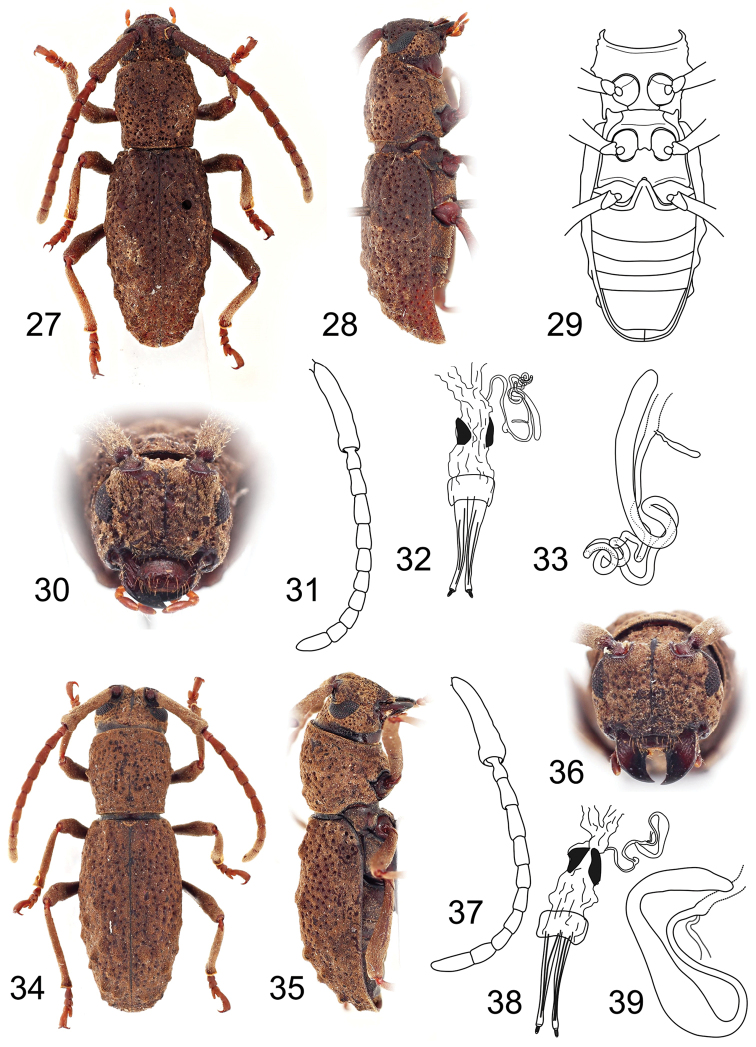
**27–33** *Microdolichostyrax hefferni* sp. n., holotype female: **27** Dorsal habitus **28** Lateral habitus **29** Ventral habitus **30** Head,frontal view **31** Antenna **32** Reproductive system **33** Spermatheca **34–39** *Microdolichostyrax minutus* sp. n., holotype female: **34** Dorsal habitus **35** Lateral habitus **36** Head, frontal view **37** Antenna **38** Reproductive system **39** Spermatheca. Not to scale.

**Figures 40–46.**
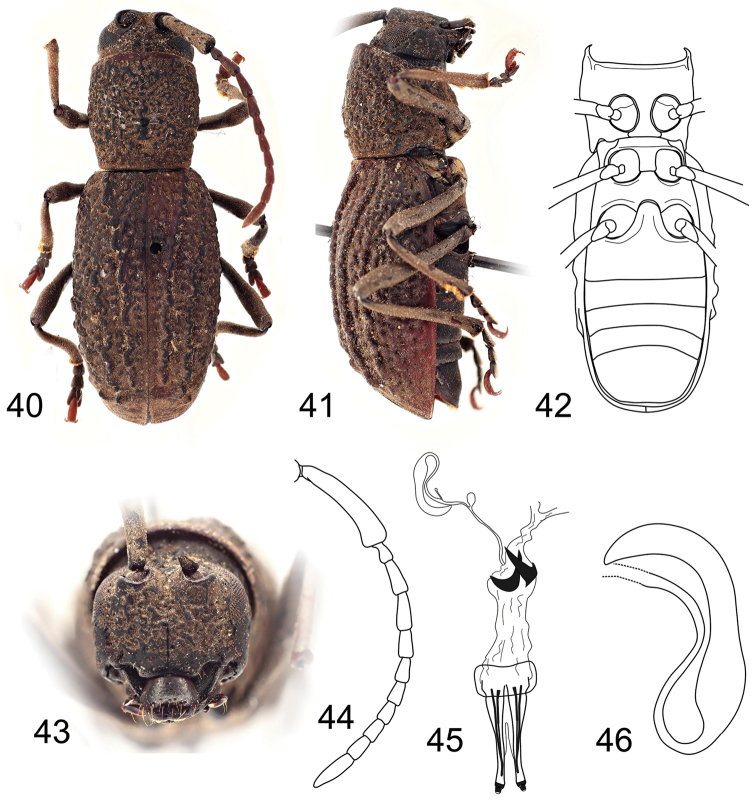
*Eurystyrax nemethi* sp. n., holotype female: **40** Dorsal habitus **41** Lateral habitus **42** Ventral habitus **43** Head, frontal view **44** Antenna **45** Reproductive system **46** Spermatheca. Not to scale.

**Figures 47–63.**
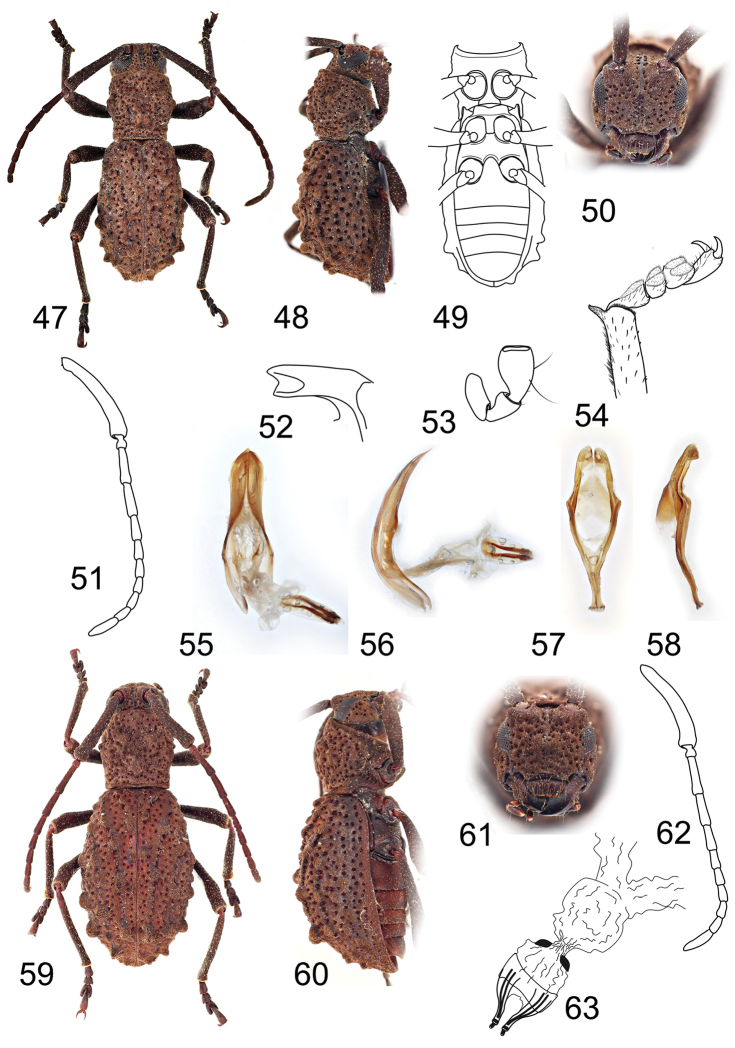
**47–58** *Borneostyrax cristatus* sp. n., holotype male: **47** Dorsal habitus **48** Lateral habitus **49** Ventral habitus **50** Head, frontal view **51** Antenna **52** Mandible apex **53** Apical maxillary palpomeres **54** Apex of protibia with protarsus **55** Penis, ventral view **56** Penis, lateral view **57** Tegmen, ventral view **58** Tegmen, lateral view **59–63** *Borneostyrax cristatus* sp. n., paratype female: **59** Dorsal habitus **60** Lateral habitus **61** Head, frontal view **62** Antenna **63** Reproductive system. Not to scale.

#### Redescription.

Body elongate to broadly oval, 9.4–11.8 mm long and 3.5–4.3 mm wide in males, and 11.1–12.5 mm long and 4.3–4.9 mm wide in females. Body coloration brown to black; antennae, palpi and legs (or only appendage joints) lighter (Figs 1–2, 13–14, 21–22). Body densely clothed with very short yellowish to light brown pubescence, incorporating fine detritus particles.

Prothorax sub-cylindrical, 0.9–1.1 times as long as wide, widest slightly before middle, then gradually narrowed towards posterior margin, laterally with one small more or less distinct tubercle; pronotal disc weakly convex, sparsely covered with deep puncturation, with more or less distinct, smooth or punctured tubercles (Figs 1, 13, 21), anterior and posterior angles obtuse. Prosternum in front of coxae 0.6–0.7 times shorter than diameter of coxal cavity, procoxal cavities circular, with lateral extension, narrowly separated. Scutellum transverse, widely rounded apically, about 3–4 times as wide as long. Elytra elongate, 1.4–1.6 times as long as wide at widest part, 1.6–1.9 times as long as pronotum in males and 1.8–2.3 times in females, basally slightly wider than posterior pronotal margin, widest near middle, from middle gradually tapered towards apex, fused along the elytral suture; each elytron with three rows of tubercles irregular in shape and size (Figs 1, 13, 21), sparsely covered by large deep punctures arranged irregularly in rows; outer elytral margin curved at lateral view (Figs 2, 14, 22). Mesoventrite with anterior edge on different plane than metaventrite; mesocoxal cavities circular, separated slightly wider than in procoxal cavities. Metaventrite transverse, more than two times as wide as long, posterior margin emarginated, with short narrow median groove. Metacoxal cavities separated as widely as mesocoxal ones, extending laterally to meet elytra. Hind wings absent. Legs long, slender; femora weakly swollen distally, tibial spurs 2-2-2, protibiae with pubescent groove (antennal cleaner) on inner face, mesotibiae with pubescent groove on outer face, metatibiae without groove; tarsal formula 4-4-4, relative lengths of metatarsomeres 1.0 : 0.7–1.0 : 1.2–1.5 : 1.8–2.4; last tarsomere with four long erected setae at ventral face, claws simple, empodium absent.

Abdomen with five ventrites (Fig. 3), first ventrite (excluding intercoxal process) almost two times longer than second; intercoxal process short, broadly rounded. Fifth ventrite with apex truncate, margin with sparse semi-erect pubescence. Male genitalia with tegmen elongate, widest before middle, basally with or without strut; parameres elongate, less than half of phallobase length, setose apically (Figs 11–12, 19–20). Penis weakly curved at lateral view, apically truncate; dorsal struts diverged from about 1/2 of penis length. Internal sac long, with paired small medial sclerites and distinct flagellar sclerites (Figs 9–10, 17–18). Female genitalia with ovipositor elongate, narrow, apically with short styli (Fig. 25). Vagina narrow, with pair of vaginal plates. Bursa copulatrix small. Spermatheca present, well-sclerotized, simple, slender, elongate, curved; sclerotized part of spermathecal duct simply coiled, distinctly shorter than spermatheca itself (Fig. 26).

#### Distribution.

Malaysia: Borneo (Sarawak: *Dolichostyrax moultoni* Aurivillius, 1911; Sabah: *Dolichostyrax longipes* Aurivillius, 1913), Indonesia (Sumatra: *Dolichostyrax basispinosus* Breuning & de Jong, 1941; Java: *Dolichostyrax tuberculatus* Fisher, 1936; *Dolichostyrax cylindricus* Breuning, 1939).

### Dolichostyrax moultoni

Taxon classificationAnimaliaColeopteraCerambycidae

Aurivillius, 1911

Figs 1–12

Dolichostyrax moultoni Aurivillius, 1911: 195.

#### Type material.

Holotype, male, “Klinkang / 1-01 // Type // NHRS-JLKB / 000022860 // 5185 / E94 + // HOLOTYPE / *Dolichostyrax* / *moultoni* Aurivillius, 1911 / Labelled by Gabriš, 2016” (NHRS).

#### Diagnosis.

This species differs from *Dolichostyrax longipes* by apex of scape without a distinct bulge (Figs 5, 16, 24), tegmen basally without distinct strut (vs. short strut in *Dolichostyrax longipes*; Figs 11, 19), and parameres with sparse long setae at apex only (vs. parameres with dense long setae at whole apical half; Figs 11, 19).

#### Redescription of holotype

(male). Body length 11.2 mm, body width 3.9 mm. Body brown; appendage joints and palpi lighter. Body densely clothed with very short golden brown pubescence, incorporating fine detritus particles. Head slightly narrower than anterior margin of pronotum. Antennae as long as body; scape gradually widened towards apex, thickest at apical part, covered with very short dense light brown pubescence; the relative ratio of antennomere lengths 2.0 : 0.2 : 1.0 : 0.8 : 0.6 : 0.4 : 0.4 : 0.4 : 0.4 : 0.5 : 0.8.

Prothorax 1.1 times as long as wide, laterally with one small obtuse tubercle; pronotal disc with a pair of distinct tubercles near middle and one median at second half; pronotal tubercles not punctured. Prosternum in front of coxae 0.7 times shorter than diameter of coxal cavity. Scutellum transverse, about four times as wide as long. Elytra elongate, 1.6 times as long as wide at widest part, 1.9 times as long as pronotum, widest before middle; each elytron with three rows of tubercles irregular in shape and size (Figs 1, 2), sparsely covered with large deep punctures arranged irregularly in rows, more distinct near elytral suture. Legs long, slender; tibial spurs with mesotibial ones inconspicuous; protarsi and left mesotarsus preserved with tarsomeres I–II only, metatarsus with tarsomere I only; relative lengths of metatarsomeres 1.0 : 1.0 : 1:3 : 2.1.

Male genitalia with tegmen elongate, widest before middle, basally without distinct strut; parameres elongate, less than half of phallobase length, apically with sparse long setae (Figs 11, 12). Penis weakly curved at lateral view, apically truncate; dorsal struts diverged from 1/2 of penis length. Internal sac long, with paired small medial and distinct flagellar sclerites (Fig. 9–10).

Female unknown.

#### Distribution.

Malaysia: Borneo (Sarawak: “Klinkang”). There is “Klinkang” written on the original label, but “Kuching” in the original description (Aurivillius 1911).

### Dolichostyrax longipes

Taxon classificationAnimaliaColeopteraCerambycidae

Aurivillius, 1913

Figs 13–26

Dolichostyrax longipes Aurivillius, 1913: 239.

#### Type material.

Holotype, male, “Batu Lawi / Expedition / Between ulu / Madihil and Lim- / bang, 5-1911 / Gazette Aug. / 1911 // NHRS-JLKB // 000022861 // 5816 /E94 + // HOLOTYPE / *Dolichostyrax* / *longipes* Aurivillius, 1913 / Labelled by Gabriš, 2016” (NHRS).

#### Other material examined.

Male, “Malaysia, Sabah / Crocker Range / I-12-2004 / Jackson coll // *Dolichostyrax* / n. sp. 1 / det. J. Sudre 06 // *Dolichostyrax* / *longipes* Aurivillius, 1913 / Gabriš det., 2016” (PCDH); male, “BORNEO, Sabah, Malaysia / Kinabalu Park, HQ / 31.1.–2.2.2000, 1500 m / lgt. Jan Cempírek // *Dolichostyrax* / *longipes* Aurivillius, 1913 / Gabriš det., 2016” (PCJC); female, “Malaysia, Sabah / Sipitang vic / II-26-2005 / local coll // *Dolichostyrax* / *longipes* Aurivillius, 1913 / Gabriš det., 2016” (PCDH); female, “Malaysia, Sabah / Ranau / II-12-2004 / Lubin coll // *Dolichostyrax* / n. sp. 1 / det. J. Sudre 06 // *Dolichostyrax* / *longipes* Aurivillius, 1913 / Gabriš det., 2016” (PCDH); female, “Malaysia, Sabah / Tenom / IV-1-2004 / local coll // *Dolichostyrax* / n. sp. / det. J. Sudre // *Dolichostyrax* / *longipes* Aurivillius, 1913 / Gabriš det., 2016” (PCDH).

#### Diagnosis.

*Dolichostyrax longipes* differs by *Dolichostyrax moultoni* by presence of a distinct bulge at apex of scape (Figs 16, 24), tegmen basally with short strut (missing in *Dolichostyrax moultoni*; Fig. 19), and parameres with with dense long setae at whole apical half (vs. setae distributed sparsely at apex of paramere only; Figs 19–20).

#### Redescription of holotype

(male). BL 9.4 mm, BW 3.5 mm. Body black, antennae and legs slightly lighter. Body densely clothed with very short golden brown pubescence, incorporating fine detritus particles. Head about as wide as anterior margin of pronotum. Antennae 1.3 times longer than body length; scape gradually only slightly widened towards apex, apical part distinctly thicker than the rest of scape, forming a distinct bulge (Fig. 16), densely covered with very short light brown pubescence; the relative ratio of antennomere lengths 2.5 : 0.3 : 1.0 : 1.0 : 0.8 : 0.7 : 0.6 : 0.6 : 0.6 : 0.6 : 0.8.

Prothorax as long as wide, laterally with one indistinct tubercle; pronotal disc with a pair of indistinct tubercles near middle and one median at second half; pronotal tubercles punctured. Prosternum in front of coxae 0.6 times shorter than diameter of coxal cavity. Scutellum transverse, more than three times as wide as long. Elytra elongate, 1.4 times as long as wide at widest part, 1.6 times as long as pronotum, widest at middle; each elytron with three rows of tubercles irregular in shape and size (Figs 13, 14), tubercles only slightly elevated from deeply wrinkled elytral surface; sparsely covered with large deep punctures arranged in rows, visible mainly from the lateral view. Legs long, slender; with all tibial spurs distinct; right protarsus and metatarsus with only tarsomere I preserved, right mesotarsus missing; relative lengths of metatarsomeres 1.0 : 0.8 : 1.2 : 1.8.

Male genitalia with tegmen elongate, widest before middle, basally with short strut; parameres elongate, less than half of phallobase length, with dense long setae at apical half (Fig. 19). Penis weakly curved at lateral view, apically truncate; dorsal struts diverged from about 1/2 of penis length. Internal sac long, with paired small medial and distinct flagellar sclerites (Figs 17–18).

#### Variability in males.

BL 9.4–11.8 mm, BW 3.5–4.3 mm. Antennae 1.0–1.3 times longer than body length. Prothorax laterally with one more or less distinct obtuse tooth; pronotal disc slightly to deeply wrinkled; pronotal and elytral tubercles more distinct in other males than holotype. Male from Kinabalu Park (PCJC) large, with pubescence very dense, yellowish brown, and with slightly narrower tegmen.

#### Description of female.

Most characters same as for males. BL 11.7–12.5 mm, BW 4.3–4.9 mm. Antennae 0.9–1.0 times longer than body length. Pronotal and elytral tubercles more or less distinct; tubercles smooth or with individual punctures. Elytra elongate, 1.4–1.6 times as long as wide at widest part, 1.8–2.3 times as long as pronotum. Female genitalia with elongate ovipositor (Fig. 25). Bursa copulatrix small. Spermatheca slender, elongate, curved; sclerotized part of spermathecal duct simply coiled, distinctly shorter than spermatheca itself (Fig. 26).

#### Distribution.

Malaysia: Borneo (Sabah).

### Genus Microdolichostyrax gen. n.

Taxon classificationAnimaliaColeopteraCerambycidae

http://zoobank.org/0F156593-8C03-452B-8F91-C4EDC18FD6DC

#### Type species.

*Microdolichostyrax hefferni* sp. n.

#### Diagnosis.

The genus *Microdolichostyrax* can be easily recognized by the following combination of characters: generally smaller habitus (BL 9.0–10.5), antennae 0.7 times as long as body, surface of scape slightly distorted (unique in Bornean Morimopsini), antennomere II 0.5–0.8 times as long as antennomere III, antennomere IV longer than antennomere III, antennomere XI longer than antennomere III, antennomeres relatively short (e.g. antennomere III length/width ratio = 1.7–1.8), mandibular apex unidentate, elytra with rows of individual tubercles, and tibial spurs 2-2-2 (Figs 27–28, 34–35).

#### Etymology.

The name *Microdolichostyrax* gen. n. refers to the smaller size of the specimens belonging to the genus, and to its similarity to *Dolichostyrax* Aurivillius. Gender: masculine.

#### Description.

Female. Body elongate, BL 9.0–10.5 mm, BW 2.9–3.7 mm. Body brown; antennae, legs and palpi lighter (Figs 27, 34). Body densely clothed with very short yellowish or chestnut brown pubescence, incorporating fine detritus particles.

Head slightly wider than anterior pronotal margin; genae convex at frontal view; frontoclypeus with midline running from interantennal groove to labrum, sparsely punctured; antennal tubercles prominent with deep depression in between; anterior margin of anteclypeus shallowly emarginate, with sparse long yellowish semi-erected setae. Labrum free, transverse, glabrous, with sparse long semi-erect setae (Figs 30, 36). Eyes rather small, reniform, vertically elongate, slightly emarginate at antennal articulations, lower lobes narrower than genae. Antennae 11-segmented, 0.7 times as long as body; scape enlarged, slightly curved, longest, reaching about half of pronotum, gradually widened towards apex, thickest at apical part, surface slightly distorted, not smooth, covered with very short dense pubescence; the rest of antennomeres with sparser pubescence, pedicel very small, shortest, the relative ratio of antennomere lengths: I–IV 3.2–3.9 : 0.5–0.8 : 1.0 : 1.1–1.3; antennomere III relatively wide (length/width ratio = 1.7–1.8), antennomere V slightly shorter than IV, antennomeres VI–X subequal in length, apical antennomere simple, 1.4–1.5 times as long as antennomere III (Figs 31, 37). Mandibles short and broad; apex unidentate (Fig. 6). Maxillary palpi 4-segmented, apical palpomere fusiform (Fig. 7). Labial palpi 3-segmented, apical palopmere fusiform.

Prothorax sub-cylindrical, 0.9–1.0 times as long as wide, widest at middle, gradually narrowed towards posterior margin, laterally with one small obtuse tubercle; pronotal disc weakly convex, sparsely covered with deep puncturation, with a pair of tubercles near middle and one median at second half; anterior and posterior angles obtuse; pronotal tubercles punctured (Figs 27, 34). Prosternum in front of coxae 0.8–0.9 times shorter than diameter of coxal cavity, procoxal cavities circular, narrowly separated (Fig. 29). Scutellum transverse, more than three times as wide as long. Elytra elongate, 1.6–1.8 times as long as wide at widest part, 2.1–2.3 times as long as pronotum, basally slightly wider than posterior pronotal margin, widest near middle, from middle gradually tapered towards apex; each elytron with three rows of irregular, slightly elevated tubercles (Figs 27, 34), sparsely covered by large deep punctures irregularly in rows, surface not wrinkled; outer elytral margin curved at lateral view (Figs 28, 35). Mesoventrite with anterior edge on different plane than metaventrite. Mesocoxal cavities circular, separated slightly wider than in procoxal cavities. Metaventrite transverse, more than two times wide as long, posterior margin emarginated, with wide, moderately deep median emargination. Metacoxal cavities separated as widely as mesocoxal ones, extending laterally to meet elytra (Fig. 29). Hind wing absent. Legs long, slender; femora weakly swollen distally, tibial spurs 2-2-2, protibiae with pubescent groove (antennal cleaner) on inner face, mesotibiae with pubescent groove on outer face, metatibiae without groove; tarsal formula 4-4-4; relative lengths of metatarsomeres 1.0 : 0.6 : 1:0 : 1.6–1.7; last tarsomere with four long erected setae at ventral face, claws simple, empodium absent.

Abdomen with five ventrites (Fig. 29), first ventrite (excluding intercoxal process) almost two times longer than second; intercoxal process short, broadly rounded. Fifth ventrite with apex truncate, margin with sparse semi-erect pubescence. Female genitalia with ovipositor elongate, narrow, apically with short styli (Figs 32, 38). Vagina narrow, with pair of vaginal plates. Bursa copulatrix small. Spermatheca present, well-sclerotized, elongate, more or less curved, apex rounded or tapered; sclerotized part of spermathecal duct short or very long, strongly coiled (Figs 33, 39).

Male unknown.

#### Distribution.

Malaysia: Borneo (Sabah).

### Microdolichostyrax hefferni sp. n.

Taxon classificationAnimaliaColeopteraCerambycidae

http://zoobank.org/FFFF7E31-3701-455A-B3F5-394D76983B7F

Figs 27–33

#### Type material.

Holotype, female, “Malaysia, Sabah / Sipitang area / II-1-2003 / local coll // *Dolichostyrax* / *longipes* / Aurivillius / det J. Sudre 06 // HOLOTYPE / *Microdolichostyrax* / *hefferni* Gabriš, Kundrata / & Trnka, 2016 / gen. et sp. n.” (HNHM, ex PCDH). Three paratypes. Female, “Malaysia, Sabah / Sipitang area / II-1-2003 / local coll // *Dolichostyrax* / *longipes* / Aurivillius / det. J. Sudre 06 // PARATYPE / *Microdolichostyrax* / *hefferni* Gabriš, Kundrata / & Trnka, 2016 / gen. et sp. n.” (PCDH); female, “Malaysia, Sabah / Mt. Trus-Madi / III-17-2003/ local coll ‘Addle‘ // *Dolichostyrax* / *longipes* / Aurivillius / det J. Sudre // PARATYPE / *Microdolichostyrax* / *hefferni* Gabriš, Kundrata / & Trnka, 2016 / gen. et sp. n.” (PCDH); female, “Malaysia, Sabah / Sipitang area / III-3-2003 / local coll ‘Unil‘ // *Dolichostyrax* / *longipes* / Aurivillius / det J. Sudre 06 // PARATYPE / *Microdolichostyrax* / *hefferni* Gabriš, Kundrata / & Trnka, 2016 / gen. et sp. n.” (PCDH).

#### Diagnosis.

This species is very similar to *Microdolichostyrax minutus* sp. n., but differs by slightly larger body (BL 9.8–10.5 mm vs. 9.0, respectively); body pubescence darker, chestnut brown (vs. yellowish brown; Figs 27, 34), antennomere II relatively longer, 0.7 times as long as antennomere III (vs. 0.5 times), spermatheca with apex rounded (vs. tapered), and the sclerotized part of spermathecal duct very long, strongly coiled (vs. short; Figs 33, 39).

#### Description of holotype

(female). BL 9.8 mm, BW 3.3 mm. Body brown; antennae, legs and palpi lighter. Body densely clothed with very short chestnut brown pubescence, incorporating fine detritus particles (Fig. 27).

Head slightly wider than anterior pronotal margin. Antennae 0.7 times as long as body; scape enlarged, reaching about half of pronotum, gradually widened towards apex, thickest at apical part, surface slightly distorted, not smooth, covered with very short dense pubescence; the relative ratio of antennomere lengths: 3.4 : 0.7 : 1.0 : 1.2 : 1.1 : 0.9 : 0.8 : 0.8 : 0.8 : 0.8 : 1.4 (Fig. 31).

Prothorax as long as wide, laterally with one small obtuse tubercle; pronotal disc with pair of tubercles near middle and one median at second half; pronotal tubercles punctured. Prosternum in front of coxae 0.8 times shorter than diameter of coxal cavity. Scutellum transverse, about 3.5 times as wide as long. Elytra elongate, 1.8 times as long as wide at widest part, 2.3 times as long as pronotum, widest near middle; each elytron with three rows of irregular, slightly elevated tubercles (Figs 27–28), sparsely covered by large deep punctures irregularly in rows, surface not wrinkled. Legs long, slender; relative lengths of metatarsomeres 1.0 : 0.6 : 1:0 : 1.6.

Female genitalia with ovipositor elongate, narrow, apically with short styli (Fig. 32). Vagina narrow, with pair of vaginal plates. Bursa copulatrix small. Spermatheca well-sclerotized, simple, slender, elongate, slightly curved, apex rounded; sclerotized part of spermathecal duct very long, strongly coiled (Fig. 33).

#### Variability.

BL 9.8–10.5 mm, BW 3.3–3.7 mm. Paratypes are slightly larger and more oval than holotype.

#### Distribution.

Malaysia: Borneo (Sabah: Sipitang, Trus Madi).

#### Etymology.

The specific name is a patronym in honor of Mr. Daniel J. Heffern (Houston, USA), who kindly provided us with the type material.

### Microdolichostyrax minutus sp. n.

Taxon classificationAnimaliaColeopteraCerambycidae

http://zoobank.org/F634EFA8-6969-43B0-A553-29BC2DB7F5C1

Figs 34–39

#### Type material.

Holotype, female, “Malaysia, Sabah / Kuamut / III-13-2014 / local coll // HOLOTYPE / *Microdolichostyrax* / *minutus* Gabriš, Kundrata / & Trnka, 2016 / sp. n. “ (HNHM, ex PCDH).

#### Diagnosis.

*Microdolichostyrax minutus* sp. n.can be recognized by the smaller body (BL 9.0 mm), body pubescence paler, yellowish brown (vs. chestnut brown in *Microdolichostyrax hefferni* sp. n.; Figs 27, 34), antennomere II 0.5 times as long as antennomere III, spermatheca with apical part tapered (vs. rounded), and the sclerotized part of spermathecal duct short, curved (vs. very long; Figs 33, 39).

#### Description of holotype

(female). BL 9.0 mm, BW 2.9 mm. Body brown; antennae, legs and palpi lighter. Body densely clothed with very short yellowish brown pubescence, incorporating fine detritus particles (Fig. 34).

Head slightly wider than anterior pronotal margin. Antennae 0.7 times as long as body; scape enlarged, reaching about half of pronotum, gradually widened towards apex, thickest at apical part, surface slightly distorted, not smooth, covered with very short dense pubescence; relative ratio of antennomere lengths: 3.2 : 0.5 : 1.0 : 1.1 : 1.0 : 0.7 : 0.8 : 0.7 : 0.7 : 0.7 : 1.5 (Fig. 37).

Prothorax 0.9 times as long as wide, widest at middle, laterally with one small obtuse tubercle; pronotal disc with pair of tubercles near middle and one median at second half; pronotal tubercles punctured. Prosternum in front of coxae 0.9 times shorter than diameter of coxal cavity. Scutellum transverse, about three times as wide as long. Elytra elongate, 1.8 times as long as wide at widest part, 2.3 times as long as pronotum, widest near middle; each elytron with three rows of irregular, slightly elevated tubercles (Figs 34–35), sparsely covered by large deep punctures irregularly in rows; surface not wrinkled. Legs long, slender; relative lengths of metatarsomeres 1.0 : 0.6 : 1:0 : 1.7.

Female genitalia with ovipositor elongate, narrow, apically with short styli (Fig. 38). Vagina narrow, with pair of vaginal plates. Bursa copulatrix small. Spermatheca present, well-sclerotized, elongate, curved, basally wider, constricted at apical 1/3, apex tapered; sclerotized part of spermathecal duct short, curved (Fig. 39).

#### Distribution.

Malaysia: Borneo (Sabah: Kuamut).

#### Etymology.

The name “*minutus*” refers to the smaller size of the species.

### Genus Eurystyrax gen. n.

Taxon classificationAnimaliaColeopteraCerambycidae

http://zoobank.org/1559EF45-63F6-4331-9931-6508C8C5A5A8

#### Type species.

*Eurystyrax nemethi* sp. n.

#### Diagnosis.

The *Eurystyrax nemethi* gen. et sp. n. can be easily recognized by its robust body (BL 14.3 mm), genae parallel-sided at frontal view (Fig. 43), elytra with distinct ridges without individual tubercles (Fig. 40), and outer elytral margin straight at lateral view (Fig. 41).

#### Description.

Female. Body robust, elongate, BL 14.3 mm, BW 5.1 mm. Body black, densely clothed with very short greyish pubescence, incorporating fine detritus particles.

Head about as wide as anterior pronotal margin, subquadrate at frontal view (genae parallel-sided); frontoclypeus with midline running from interantennal groove to labrum, sparsely punctured; antennal tubercles prominent with deep depression in between; anterior margin of anteclypeus shallowly emarginate, with sparse long yellowish semi-erected setae (Fig. 43). Labrum free, transverse, glabrous, with sparse long erected setae at apical half; frontal margin with very short dense golden pubescence. Eyes rather small, reniform, vertically elongate, slightly emarginate at antennal articulations, lower lobes distinctly narrower than genae. Antennae 11-segmented, 0.6 times as long as body; scape enlarged, slightly curved, longest, reaching about half of pronotum, gradually widened towards apex, thickest at apical part, surface smooth, covered with very short dense pale pubescence; the rest of antennomeres with sparser pubescence, pedicel very small, shortest, the relative ratio of antennomere lengths: 3.4 : 0.4 : 1.0 : 1.1 : 0.8 : 0.8 : 0.7 : 0.6 : 0.6 : 0.7 : 1.4 (Fig. 44), antennomere III 2.4 times as long as wide. Mandibles short and broad, apex unidentate (Fig. 6). Maxillary palpi 4-segmented, apical palpomere fusiform. Labial palpi 3-segmented, apical palpomere of same shape as maxillary one.

Prothorax sub-cylindrical, as long as wide, widest at middle, gradually slightly narrowed towards posterior margin, laterally without tubercles; pronotal disc sub-parallel sided, weakly convex, surface coarsely wrinkled, without distinct tubercles, sparsely covered with deep puncturation, anterior and posterior angles obtuse (Fig. 40). Prosternum in front of coxae 0.8 times shorter than diameter of coxal cavity, procoxal cavities circular, narrowly separated (Fig. 42). Scutellum transverse, about four times as wide as long. Elytra elongate, sub-parallel, 1.7 times as long as wide at widest part, 2.2 times as long as pronotum, basally slightly wider than posterior pronotal margin, widest near middle, from middle gradually slightly tapered towards apex; each elytron with three elevated ridges, without individual tubercles, sparsely covered with deep punctures arranged in rows; outer elytral margin straight at lateral view (Fig. 41). Mesoventrite with anterior edge on different plane than metaventrite. Mesocoxal cavities circular, separated slightly wider than in procoxal cavities. Metaventrite transverse, more than 2.5 times wide as long, posterior margin emarginated, with wide moderately deep median emargination. Metacoxal cavities separated slightly wider than mesocoxal ones, extending laterally to meet elytra (Fig. 42). Hind wing absent. Legs long, slender; femora weakly swollen distally, not reaching elytral apex; tibial spurs 2-2-2, protibiae with pubescent groove (antennal cleaner) on inner face, mesotibiae with pubescent groove on outer face, metatibiae without groove; tarsal formula 4-4-4; relative lengths of metatarsomeres 1.0 : 0.7 : 1:0 : 1.5; last tarsomere with four long erected setae at ventral face, claws simple, empodium absent.

Abdomen with five ventrites; first ventrite (excluding intercoxal process) more than 1.5 times longer than second; intercoxal process short, broadly rounded (Fig. 42). Fifth ventrite with apex truncate, margin with sparse semi-erect pubescence. Female genitalia with ovipositor elongate, narrow, apically with short styli (Fig. 45). Vagina narrow, with pair of vaginal plates. Bursa copulatrix small. Spermatheca present, well-sclerotized, simple, elongate, slightly curved, widened basally; sclerotized part of spermathecal duct simple, short (Fig. 46).

Male unknown.

#### Etymology.

The name *Eurystyrax* is a combination of words “eury” (referring to the wide habitus of the holotype) and “styrax” (part of the generic name *Dolichostyrax*). Gender: masculine.

### Eurystyrax nemethi sp. n.

Taxon classificationAnimaliaColeopteraCerambycidae

http://zoobank.org/87E1ED94-FFD2-47CE-AFB6-DA44C96B0BE4

Figs 40–46

#### Type material.

Holotype, female, “Nord-Borneo / Kinabalu, West- / hang, ca 2800 m // 4.III.1969 / Dr. H. Löffler leg. // *Dolichostyrax* / *longipes* Aur. / det. Breuning 72. // HOLOTYPE / *Eurystyrax* / *nemethi* Gabriš, Kundrata / & Trnka, 2016 / gen. et sp. n. “ (HNHM).

#### Description of holotype

(female). BL 14.3 mm, BW 5.1 mm. Body black, densely clothed with very short greyish pubescence, incorporating fine detritus particles.

Head about as wide as anterior pronotal margin, subquadrate at frontal view (genae parallel-sided); frontoclypeus sparsely punctured; anterior margin of anteclypeus shallowly emarginate, with sparse long yellowish semi-erected setae (Fig. 43). Labrum transverse, glabrous, with sparse long erected setae at apical half; frontal margin with very short dense golden pubescence. Eyes rather small, reniform, slightly emarginate at antennal articulations, lower lobes distinctly narrower than genae. Antennae 0.6 times as long as body; scape enlarged, slightly curved, longest, reaching about half of pronotum, gradually widened towards apex, surface smooth, covered with very short dense pale pubescence; the rest of antennomeres with sparser pubescence, pedicel very small, shortest, the relative ratio of antennomere lengths: 3.4 : 0.4 : 1.0 : 1.1 : 0.8 : 0.8 : 0.7 : 0.6 : 0.6 : 0.7 : 1.4 (Fig. 44), antennomere III 2.4 times as long as wide. Mandibles short and broad, apex unidentate (Fig. 6). Maxillary and labial palpi with apical palpomere fusiform.

Prothorax as long as wide, widest at middle, laterally without tubercles; pronotal disc sub-parallel sided, weakly convex, surface coarsely wrinkled, without distinct tubercles, sparsely covered with deep puncturation, anterior and posterior angles obtuse (Fig. 40). Scutellum about four times as wide as long. Elytra elongate, sub-parallel, widest near middle, from middle gradually slightly tapered towards apex; each elytron with three elevated ridges, without individual tubercles, sparsely covered with deep punctures arranged in rows; outer elytral margin straight at lateral view (Fig. 41). Legs long, slender; tibial spurs 2-2-2, tarsal formula 4-4-4; relative lengths of metatarsomeres 1.0 : 0.7 : 1:0 : 1.5.

Abdomen with fifth ventrite truncate apically, margin with sparse semi-erect pubescence. Female genitalia with ovipositor elongate, narrow, apically with short styli (Fig. 45). Vagina narrow, with pair of vaginal plates. Bursa copulatrix small. Spermatheca present, well-sclerotized, simple, elongate, slightly curved, widened basally; sclerotized part of spermathecal duct simple, short (Fig. 46).

Male unknown.

#### Distribution.

Malaysia: Borneo (Sabah: Kinabalu).

#### Etymology.

This species is named after Mr. Tamás Németh (HNHM, Budapest, Hungary).

### Genus Borneostyrax gen. n.

Taxon classificationAnimaliaColeopteraCerambycidae

http://zoobank.org/0D57C7D9-A3A5-4435-ACF5-9908E0DD10F7

#### Type species.

*Borneostyrax cristatus* sp. n.

#### Diagnosis.

This genus is unique within Bornean Morimopsini by having bidentate mandibular apex (Fig. 52) and elytra with tubercles forming distinct ridges (Figs 48, 60) in both sexes; tibial spurs 0-0-2, distinct protrusions on apices of protibiae and mesotibiae (Fig. 54), and terminal maxillary and labial palpomeres widened, flattened and truncate in males (Fig. 53), and short, wide ovipositor, large sac-like vagina and missing spermatheca in females (Fig. 63).

#### Description.

Body robust, elongate, 10.8 mm long and 3.9 mm wide in male, and 12.6–14.6 mm long and 4.9–5.5 mm wide in females. Body reddish brown to dark brown; appendage joints lighter, palpi brown to black. Body very densely clothed with very short golden brown pubescence; scape, legs, scutellum, apex of elytra and abdominal ventrites covered with longer sparse semi-erected yellow setae (Fig. 47).

Head about as wide as anterior margin of pronotum; genae convex at frontal view; frontoclypeus with distinct midline running from interantennal groove to labrum, sparsely punctured, punctures deep with setae inside; antennal tubercles prominent with moderately deep depression in between; antennal cavities opened dorsally; anterior margin of anteclypeus shallowly emarginate, with sparse long yellowish semi-erected setae. Labrum free, transverse, glabrous, covered with long, sparse semi-erect setae, apical margin with short dense pubescence (Fig. 50). Eyes moderately-sized, vertically elongate, emarginate at antennal articulations, lower lobes slightly narrower than genae. Antennae 11-segmented, about as long as body in male and 0.8–0.9 times in females; scape enlarged, slightly curved, longest, reaching the second half of pronotum, gradually widened towards apex, thickest at apical part, with sparse yellow semi-erect setae, the rest of antennomeres with much sparser and thinner setae, pedicel very small, shortest, the relative length ratio of antennomeres I–IV 2.4–2.9 : 0.2–0.3 : 1.0 : 0.9–1.0; antennomere III relatively narrow (length/width ratio = 3.4–3.6; Fig. 51); antennomere V slightly shorter than antennomere IV, antennomeres VI–X subequal in length, antennomere XI shorter than antennomere III. Mandibles short and broad; apex bidentate (Fig. 52). Maxillary palpi 4-segmented, ultimate palpomere with apical half widened, flattened, apex truncate in males; ultimate palpomere fusiform in females (Fig. 53). Labial palpi 3-segmented, ultimate palpomere with apical half widened, flattened, apex truncate in males; ultimate palpomere fusiform in females.

Prothorax sub-cylindrical, as long as wide, widest at middle, laterally with one small obtuse tubercle; pronotal disc weakly convex, sparsely covered with deep puncturation, with pair of more or less distinct tubercles near middle and two median at first and second half, respectively; pronotal tubercles smooth or sparsely punctured; anterior and posterior angles obtuse. Prosternum in front of coxae 0.7 times shorter than diameter of coxal cavity, sparsely punctured; procoxal cavities circular, narrowly separated (Fig. 49). Scutellum transverse, about three times as wide as long. Elytra elongate, 1.6–1.7 times as long as wide at widest part, 2.1–2.5 times as long as pronotum, basally wider than posterior pronotal margin, widest slightly after middle, then gradually tapered towards apex, fused along the elytral suture; each elytron with three rows of prominent irregular tubercles forming distinct ridges (Figs 47–48), sparsely covered with large deep punctures located irregularly in rows; elytra covered with very dense short pubsecence, apically with sparse long erected yellowish brown setae; outer elytral margin curved at lateral view (Fig. 48). Mesoventrite with anterior edge on different plane than metaventrite. Both mesoventrite and metaventrite without puncturation. Mesocoxal cavities circular, separated wider than in procoxal cavities. Metaventrite transverse, more than two times wider than long, posterior margin emarginated, with short narrow median emargination. Metacoxal cavities separated slightly wider than in mesocoxal ones, extending laterally to meet elytra (Fig. 49). Hind wing absent. Legs long, slender; femora weakly swollen distally, tibial spurs 0-0-2 in male, 2-2-2 in females, protibiae with pubescent groove (antennal cleaner) on inner face, inner face apically prolonged forming distinct, wide, gradually tapered protrusion in male (Fig. 54), simple in females; mesotibiae with pubescent groove on outer face, inner face with distinct protrusion as in protibiae but shorter in male, simple in female; metatibiae without groove, without protrusion; tarsal formula 4-4-4, relative lengths of metatarsomeres 1.0 : 0.5–0.6 : 0.8–0.9 : 1.2–1.7; last tarsomere with four long erected setae at ventral face, claws simple, empodium absent.

Abdomen with five ventrites; first ventrite (excluding intercoxal process) about or slightly more than 1.5 times longer than second; intercoxal process short, broadly rounded. Fifth ventrite with apex broadly rounded in male, truncate in females, margin with very sparse semi-erect pubescence. Male genitalia with tegmen elongate, widest at posterior 1/3, basally with long strut; parameres short, distinctly shorter than phallobase, apically with short fine setae (Figs 57–58). Penis weakly curved at lateral view, apically acuminate; dorsal struts diverged before 1/2 of penis length. Internal sac moderately long, with paired short medial and distinct flagellar sclerites (Figs 55–56). Female genitalia with ovipositor short, wide, apically with short styli (Fig. 63). Vagina sac-like, large, with pair of vaginal plates. Spermatheca absent.

#### Etymology.

The name *Borneostyrax* is a combination of words “Borneo” (geographical origin of the genus) and “styrax” (part of the generic name *Dolichostyrax*). Gender: masculine.

### Borneostyrax cristatus sp. n.

Taxon classificationAnimaliaColeopteraCerambycidae

http://zoobank.org/18C7327E-8B12-4073-AFEC-A109D89E4665

Figs 47–63 , 64–67

#### Type material.

Holotype, male, “Malaysia, Sabah / Tenom / III-12-2008 / local coll // *Dolichostyrax* / *moultoni* / Aurivillius / det J. Sudre 06 // HOLOTYPE / *Borneostyrax* / *cristatus* Gabriš, Kundrata / & Trnka, 2016 / gen. et sp. n. “ (HNHM, ex PCDH). Three paratypes. Female, “Malaysia, Sabah / Crocker Range, vic. / Trus Madi, III-26- / 2000 local coll. // PARATYPE / *Borneostyrax* / *cristatus* Gabriš, Kundrata / & Trnka, 2016 / gen. et sp. n. “ (PCDH); female, “Malaysia, Sabah / Tongod 500m / III-18-2014 / local coll // PARATYPE / *Borneostyrax* / *cristatus* Gabriš, Kundrata / & Trnka, 2016 / gen. et sp. n. “ (PCDH); female, “Malaysia: Sabah / Crocker Range / 10 February 2003 / LG Bezark, collection // PARATYPE / *Borneostyrax* / *cristatus* Gabriš, Kundrata / & Trnka, 2016 / gen. et sp. n. “ (PCLB).

#### Other material examined.

Female, “Malaysia, Sabah / Sipitang area / IV-11-2002 / local coll ‘Unil‘ // *Dolichostyrax* / *moultoni* / Aurivillius / det J. Sudre // *Borneostyrax* / *cristatus* Gabriš, Kundrata / & Trnka, 2016 / Gabriš det., 2016” (PCDH).

#### Description of holotype

(male). BL 10.8 mm, BW 3.9 mm. Body dark brown; appendage joints lighter, palpi black. Body very densely clothed with very short golden brown pubescence; scape, legs, scutellum, apex of elytra and abdominal ventrites covered with longer sparse semi-erected yellow setae (Fig. 47).

Head about as wide as anterior margin of pronotum; genae convex at frontal view; frontoclypeus with distinct midline running from interantennal groove to labrum, sparsely punctured; anterior margin of anteclypeus shallowly emarginate, with sparse long yellowish semi-erected setae. Labrum transverse, glabrous, covered with long, sparse semi-erect setae, apical margin with short dense pubescence (Fig. 50). Eyes moderately-sized, vertically elongate, emarginate at antennal articulations, lower lobes slightly narrower than genae. Antennae about as long as body; scape enlarged, slightly curved, longest, reaching the second half of pronotum, gradually widened towards apex, with sparse yellow semi-erect setae, the rest of antennomeres with much sparser and thinner setae, pedicel very small, shortest, the relative ratio of antennomere lengths 2.9 : 0.3 : 1.0 : 1.0 : 0.8 : 0.6 : 0.6 : 0.5 : 0.5 : 0.6 : 0.8, antennomere III relatively narrow (length/width ratio = 3.4–3.6; Fig. 51). Mandibles short and broad; apex bidentate (Fig. 52). Maxillary palpi and labial palpi with ultimate palpomere widened apically, flattened, apex truncate (Fig. 53).

Prothorax sub-cylindrical, as long as wide, widest at middle, laterally with one small obtuse tubercle; pronotal disc weakly convex, sparsely covered with deep puncturation, with pair of distinct tubercles near middle and two median at first and second half, respectively; pronotal tubercles sparsely punctured; anterior and posterior angles obtuse. Scutellum transverse, about three times as wide as long. Elytra elongate, 1.7 times as long as wide at widest part, 2.1 times as long as pronotum; each elytron with three rows of prominent irregular tubercles forming distinct ridges (Figs 47–48), sparsely covered with large deep punctures located irregularly in rows; elytra covered with very dense short pubsecence, apically with sparse long erected yellowish brown setae; outer elytral margin curved at lateral view (Fig. 48). Legs long, slender; femora weakly swollen distally, tibial spurs 0-0-2, tarsal formula 4-4-4, relative lengths of metatarsomeres 1.0 : 0.5 : 0.8 : 1.4.

Abdomen with five ventrites; first ventrite (excluding intercoxal process) about 1.5 times longer than second; intercoxal process short, broadly rounded. Fifth ventrite with apex broadly rounded, margin with very sparse semi-erect pubescence. Male genitalia with tegmen elongate, widest at posterior 1/3, basally with long strut; parameres short, distinctly shorter than phallobase, apically with short fine setae (Figs 57–58). Penis weakly curved at lateral view, apically acuminate; dorsal struts diverged before 1/2 of penis length. Internal sac moderately long, with paired short medial and distinct flagellar sclerites (Figs 55–56).

#### Description of female.

Most characters same as for males. BL 12.6–14.6 mm, BW 4.9–5.5 mm. Body reddish brown to brown; appendage joints lighter, palpi brown. Antennae 0.8–0.9 times as long as body length. Maxillary and labial palpi with ultimate palpomeres fusiform (Fig. 7). Pronotal tubercles less distinct; tubercles smooth or with individual punctures. Elytra elongate, 1.6–1.7 times as long as wide at widest part, 2.1–2.5 times as long as pronotum. Legs long, slender; tibial spurs 2-2-2; protibiae and mesotibiae without wide apical protrusions on inner faces, relative lengths of metatarsomeres 1.0 : 0.5–0.6 : 0.8–0.9 : 1.2–1.7. Abdomen with first ventrite (excluding intercoxal process) more than 1.5 times longer than second; fifth ventrite with apex truncate. Female genitalia with ovipositor short, wide, apically with short styli (Fig. 63). Vagina sac-like, large, with pair of vaginal plates. Spermatheca absent.

#### Remark.

**Figures 64–67.**
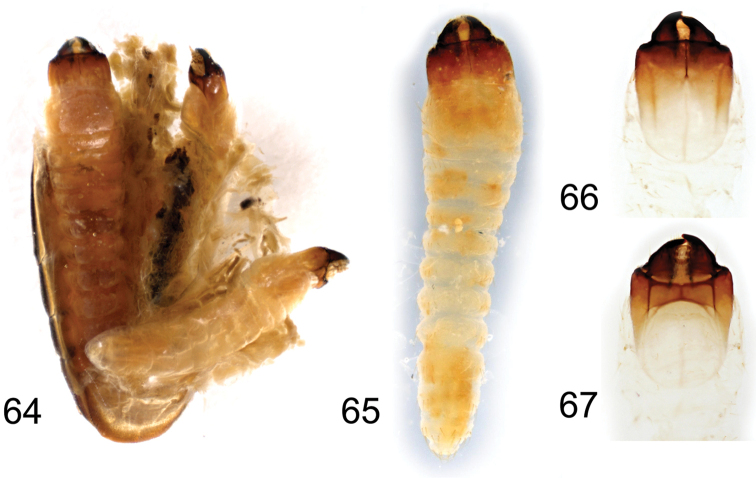
*Borneostyrax cristatus* sp. n., larvae from one of the paratype females: **64** Separated and partially opened female abdomen with three larvae, dorsal view **65** Larva, dorsal habitus **66** Larval head capsule, dorsal view **67** Larval head capsule, ventral view. Not to scale.

Two females contained large larvae (two and three, respectively) inside their abdomens. The larvae filled most of the females’ abdomens and were located with their heads oriented towards the abdominal base (Fig. 64). Apparently, there were thin egg shells at least partly covering the larvae, but unfortunately, we were not able to specify where exactly in internal genitalia were larvae localized due to the partly damaged thin membranous structures inside the female internal reproductive organs. This damage was caused by the dissection because of two factors - first, the presence of larvae in the female abdomen was an unexpected finding as (ovo)viviparity has not been reported for any long-horned beetle to date, and second, it was studied in dry material, re-moistened only before the dissection.

#### Description of larva.

Body up to 7.0 mm long and 1.6 mm wide, elongate, subcylindrical, creamy white, heavily sclerotized head capsule and mandibles darker (Fig. 65). Head capsule (Figs 66–67) 1.7 mm long and 1.3 mm wide, prognathous; anterior margin of cranium with long erect setae; medial endocarina extending to clypeus. Clypeus membranous, broad, trapezoidal. Labrum free, broadly rounded apically, sparsely setose. Antennae very small, terminal antennomere reduced, narrow. Mandibles broad, slightly curved, basally with long sparse setae. Maxillary palpi 3-segmented, apical palpomere elongate, narrow, longer than palpomere II. Labial palpi 2-segmented. Legs absent. Thoracic and abdominal segments not sclerotized, laterally sparsely setose; last two segments bearing also long erect setae dorsally.

#### Distribution.

Malaysia: Borneo (Sabah).

#### Etymology.

The specific name refers to the distinct ridges of tubercles on elytra (Fig. 48).

### Identification key to the genera of Bornean Morimopsini and species of *Dolichostyrax* Aurivillius, *Borneostyrax* gen. n., *Microdolichostyrax* gen. n. and *Eurystyrax* gen. n.

**Table:** 

1	Antennomere II distinctly longer than antennomere III	***Anexodus* Pascoe, 1886**
–	Antennomere II shorter than antennomere III	**2**
2	Body slender, narrow, parallel-sided; BL/BW = 3.5; tibial spurs 1-1-2; elytral apex truncate; elytral tubercles only at apical half (females unknown)	***Pantilema* Aurivillius**
–	Body more robust, mostly broadly oval; BL/BW = 2.4–3.1; tibial spurs 0-0-2 or 2-2-2; elytral apex rounded; elytral tubercles distributed along whole elytral length and/or forming distinct ridges	**3**
3	Antennomere III shorter than antennomere XI; antennomere II 0.4–0.7 times as long as antennomere III; antennomere III 1.7–2.4 times longer than wide	**4**
–	Antennomere III longer than antennomere XI; antennomere II 0.2–0.3 times as long as antennomere III; antennomere III 3.2–4.1 times longer than wide	**6**
4	Body larger (BL 14.3 mm); genae parallel-sided at frontal view (Fig. 43); surface of scape smooth; elytra with distinct ridges without individual tubercles (Fig. 40); outer margin straight at lateral view (Fig. 41)	**(*Eurystyrax* gen. n.) *Eurystyrax nemethi* sp. n.**
–	Body smaller (BL 9.0–10.5 mm); genae convex at frontal view (Figs 30, 36); surface of scape slightly distorted; elytra with rows of individual tubercles (Figs 27, 34); outer margin curved at lateral view (Figs 28, 35)	**(*Microdolichostyrax* gen. n.) 5**
5	Body pubescence darker, chestnut brown; antennomere II 0.7 times as long as antennomere III; spermatheca with apex rounded; sclerotized part of spermathecal duct very long, strongly coiled (Fig. 33)	***Microdolichostyrax hefferni* sp. n.**
–	Body pubescence paler, yellowish brown; antennomere II 0.5 times as long as antennomere III; spermatheca with apex tapered; sclerotized part of spermathecal duct short, curved (Fig. 39)	***Microdolichostyrax minutus* sp. n.**
6	Mandibular apex bidentate (Fig. 52); elytra with tubercles forming distinct ridges (Figs 48, 60); tibial spurs 0-0-2 in male; protibiae and mesotibiae apically with distinct protrusions in male (Fig. 54); terminal maxillary and labial palpomeres widened, flattened and truncate in male (Fig. 53); metatarsomere III 0.8–1.0 times as long as metatarsomere I	**(*Borneostyrax* gen. n.) *Borneostyrax cristatus* sp. n.**
–	Mandibular apex unidentate (Fig. 6); elytra with rows of individual tubercles (Figs 1, 13); tibial spurs 2-2-2 in male; protibiae and mesotibiae apically without distinct protrusions in male; terminal maxillary and labial palpomeres fusiform in male (Fig. 7); metatarsomere III 1.2–1.5 times as long as metatarsomere I	**(*Dolichostyrax* Aurivillius) 7**
7	Apex of scape thickened moderately (Fig. 5); tegmen basally without distinct strut; parameres with sparse long setae at apex only (Fig. 11)	***Dolichostyrax moultoni* Aurivillius**
–	Apex of scape thickened substantially, forming distinct bulge (Figs 16, 24); tegmen basally with short strut; parameres with dense long setae at apical half (Fig. 19)	***Dolichostyrax longipes* Aurivillius**

## Discussion

### Diversity of *Morimopsini* in Borneo

Borneo is one of the major biodiversity hotspots in the world (de Bruyn et al. 2014) and especially mountain ranges of north-eastern Borneo, which is the presumed Pleistocene rainforest refugium, host numbers of endemic organisms (e.g. Gathorne-Hardy et al. 2002, Merckx et al. 2015). This is also the case for the flightless Bornean long-horned beetle genera classified in Morimopsini (Breuning 1950), which are distributed almost exclusively in the mountain regions of Sabah, where the endemism appears to be highest (Gathorne-Hardy et al. 2002). This is, however, challenged by some recent studies, which pointed out that the northern parts of the island are just incomparably better sampled than the interior Borneo (see Beck and Rüdlinger 2014 for a review). Therefore, it is not clear whether the current northern distribution of Morimopsini in Borneo is caused by an influence of the Pleistocene refugial history or fact, that no material is known from the Indonesian part of the island which is hardly accessible to scientific exploration.

The cerambycid tribe Morimopsini contains many morphologically distinct lineages, and its limits and classification are in deep need of revision (Breuning 1950, Sudre 1997). Because the higher lamiine classification is beyond the scope of this paper, we retain using the Breuning’s (1950) concept of the tribe with the inclusion of the Bornean genera *Dolichostyrax*, *Anexodus*, and *Pantilema*. The specimens of Morimopsini are rarely collected, probably due to their cryptic life in the tropical forest litter and highly restricted vagility caused by the absence of wings. Therefore, we had only a limited number of specimens available for our study, but in spite of it, our study revealed that this group is much more speciose than previously believed. We found surprisingly high morphological diversity in the Bornean Morimopsini, which resulted in the descriptions of three new genera with four new species. Given their limited distributional ranges in stable long-term habitats of humid mountain forests together with the high speciation rates known for the flightless lineages (Ikeda et al. 2012, Vogler and Timmermans 2012), the high diversity found in the studied genera is not such surprising. Considering the rarity of Morimopsini specimens in the collections, their life-history and hitherto unexplored areas in Borneo, we can expect many more species will be discovered in that island in the near future.

### First case of (ovo)viviparity in Cerambycidae

The vast majority of insects are oviparous, i.e. their females lay eggs and embryogenesis occurs after oviposition. Ovoviviparous species retain their eggs in the genital tracts until the larvae are ready to hatch. There are no special nutritional adaptations developed in egg or female’s body; embryo uses only nutritional reserves from the egg cytoplasm. On the other hand, in truly viviparous species the embryo receives nourishment also (or only) from the parent. The ovoviviparity is sometimes considered as a transitional stage between oviparity and viviparity, but also very often treated as a special case of viviparity (Hagan 1951, Iwan 2000, Gullan and Cranston 2014). The (ovo)viviparous reproduction is a relatively rare phenomenon in insects and occurs in some Ephemeroptera, Dermaptera, Blattodea, Plecoptera, Psocodea, Thysanoptera, Homoptera, Neuroptera, Coleoptera, Strepsiptera, Hymenoptera, Diptera, Trichoptera and Lepidoptera (e.g. Hagan 1951, Michaelis 1984, Meier et al. 1999, Iwan 2000, Heppner 2009, Kočárek 2009). However, the reproductive strategies of many insect lineages remain unknown, and the viviparity might be in fact much more common.

Within Coleoptera, viviparity (in all cases as ovoviviparity) has been reported only for the several phylogenetically unrelated families – adephagan Carabidae (Liebherr and Kavanaugh 1985) and polyphagan Staphylinidae (Aleocharinae; Schiødte 1853), Chrysomelidae (Chrysomelinae: Chrysomelini; Perroud 1855, Bontems 1984), Micromalthidae (Barber 1913), and Tenebrionidae (Tenebrioninae: Pedinini and Ulomini; Tschinkel 1978, Iwan 2000, Dutrillaux et al. 2010). Here we add also Cerambycidae to the list of beetle families for which some (ovo)viviparous species are known. In Chrysomelidae, which are phylogenetically related to Cerambycidae (see e.g. McKenna et al. 2015), ovoviviparous females are characterized by the loss of spermatheca and the first instar larvae by the loss of eggbursters (Reid 2014). Another morphological feature associated with ovoviviparity is shortened ovipositor, which is more adapted to laying large eggs or to larviposition (Meier et al. 1999, Iwan 2000). Indeed, the females of *Borneostyrax* gen. n. have genitalia with short, wide ovipositor and without spermatheca (Fig. 63). In two females out of four, we found relatively large larvae (two and three, respectively) without any visible eggbursters (Fig. 65). These morphological features, which are present exclusively in this genus, clearly indicate the presence of (ovo)viviparity in *Borneostyrax* gen. n. This phenomenon is commonly associated with parthenogenesis in Chrysomelidae; however, we have a male associated with females in *Borneostyrax* gen. n. and absolutely no information on the life-history for this lineage. Further detailed study of more material is needed for the better understanding of the reproductive strategy in this genus.

## Supplementary Material

XML Treatment for Dolichostyrax

XML Treatment for Dolichostyrax moultoni

XML Treatment for Dolichostyrax longipes

XML Treatment for Microdolichostyrax

XML Treatment for Microdolichostyrax hefferni

XML Treatment for Microdolichostyrax minutus

XML Treatment for Eurystyrax

XML Treatment for Eurystyrax nemethi

XML Treatment for Borneostyrax

XML Treatment for Borneostyrax cristatus
